# Clinical management of seronegative and seropositive rheumatoid arthritis: A comparative study

**DOI:** 10.1371/journal.pone.0195550

**Published:** 2018-04-06

**Authors:** Sang-Tae Choi, Kwang-Hoon Lee

**Affiliations:** 1 Department of Internal Medicine, Chung-Ang University College of Medicine, Seoul, South Korea; 2 Department of Internal Medicine, Dongguk University Ilsan Hospital, Goyang, South Korea; Mie Daigaku, JAPAN

## Abstract

Both rheumatoid factor (RF) and anti-cyclic citrullinated peptide antibody (ACPA) are associated with poor radiologic outcomes in patients with rheumatoid arthritis (RA). In general, RA patients positive for RF or ACPA (SPRA) are considered to manifest an aggressive disease course compared with seronegative RA patients (SNRA). However, the relationship between seropositivity and measures of disease severity other than radiologic outcome is disputed. In this study, we sought to compare the clinical presentations and treatment outcomes of SNRA and SPRA patients. A total of 241 patients diagnosed with DMARD-naïve RA under either 1987 American College of Rheumatology (ACR) criteria or 2010 ACR/European League Against Rheumatism (EULAR) criteria were identified (40 with SNRA and 201 with SPRA). We investigated the disease activity measures including ESR, CRP, patient VAS, 28 tender/swollen joint count (28 TJC, 28 SJC) and DAS28 as well as radiologic outcomes at baseline, 1 and 2 years after conventional treatment with DMARD. Age, sex and disease duration were similar between SNRA and SPRA. However, the baseline 28 TJC (4.7±2.9 vs. 3.3±2.7, *p* = 0.004), 28 SJC (4.3±3.0 vs. 2.9±2.3, *p* = 0.001) and DAS28 (5.1±1.0 vs. 4.7±1.0, *p* = 0.043) components were significantly higher in SNRA than in SPRA. Over 2 years of similar treatment with DMARDs, all disease activity measures significantly improved in both groups. Comparison among populations matched for baseline disease activity showed that ΔDAS28 at 1 year was greater in SNRA than in SPRA (-2.84±1.32 vs. -3.70±1.29, *p* = 0.037) in high disease activity population (DAS28-ESR>5.1). Radiologic outcomes at baseline and at 1- or 2-year follow-up were similar between the 2 groups. In conclusion, SNRA patients manifested more active disease at baseline, but showed a better response to treatment compared with SPRA. SNRA does not appear to be a benign subtype of RA.

## Introduction

Rheumatoid factor (RF) is an autoantibody, which was first detected in rheumatoid arthritis (RA), and has been used in the diagnosis of RA. Anti-cyclic citrullinated peptide antibody (ACPA) has gained much attention recently, as a valuable marker in diagnosing and predicting the prognosis of RA [1]. It is as sensitive as, but much more specific than RF in diagnosing RA [2]. In addition, it plays an important role in the pathogenesis of RA and is significantly associated with radiographic progression [3]. In this sense, RF and ACPA have been regarded as poor prognostic markers of RA, and are used as evidence to justify intensive treatment in seropositive RA patients (SPRA) [4]. However, it is uncertain whether patients with SPRA manifest worse disease course compared with seronegative RA patients (SNRA) in disease activity measures other than radiologic outcome. Studies reported greater severity of disease and impaired function in SPRA patients both during disease presentation and after treatment with disease-modifying anti-rheumatic drugs (DMARDs) [5, 6]. By contrast, other studies reported that SNRA patients had more severe inflammatory activity compared to SPRA assessed clinically and by ultrasound [7] and that SNRA patients showed worse radiographic outcome compared with SPRA [8]. This divergence may be attributed to differences in the patient populations selected, inclusion criteria and measures of disease activity among studies. In this study, we investigated and compared the clinical presentations and treatment outcomes among patients diagnosed with SPRA and SNRA in a real-world setting.

## Materials and methods

### Study population

**Fig 1:**
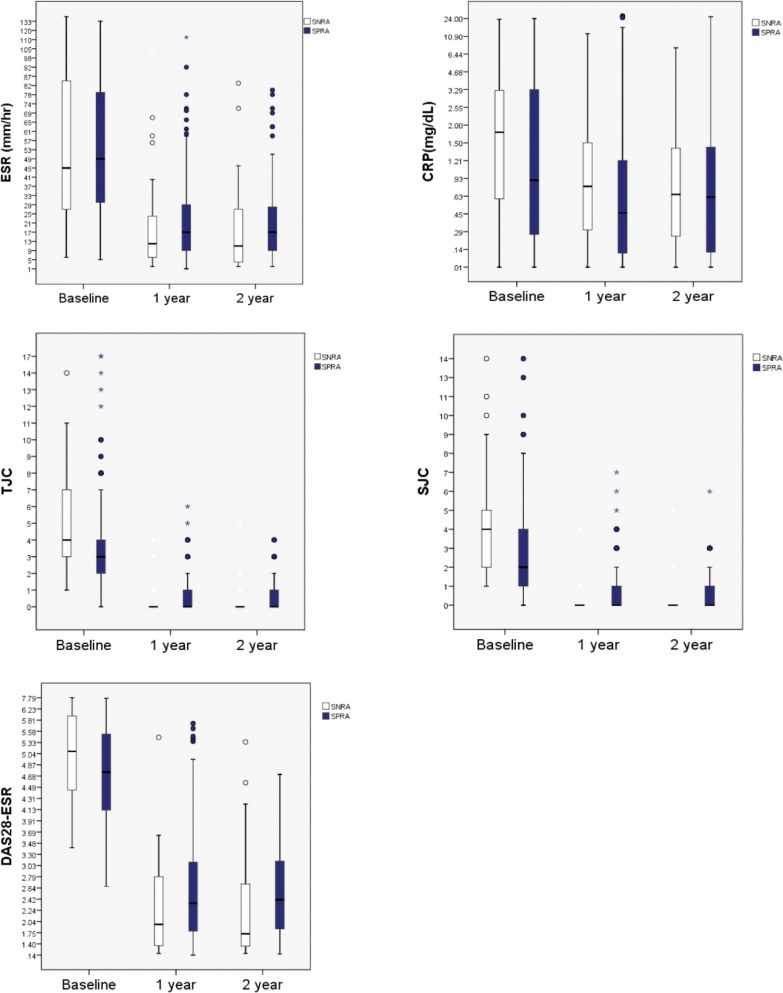
Flow diagram of the study design.

We identified a total of 241 patients with DMARD-naïve RA diagnosed according to the 1987 American College of Rheumatology (ACR) criteria [9] or the 2010 ACR/European League Against Rheumatism (EULAR) criteria [10], who attended the rheumatology clinic in Chung-Ang University Hospital in Seoul, Korea and Dongguk University Ilsan Hospital in Goyang, Korea from March 2011 to May 2017. Patients with the following conditions were excluded from analysis: 1) patients younger than 18 years; 2) patients who were already taking DMARDs at the time of first visit; and 3) patients with undifferentiated arthritis not meeting 1987 or 2010 criteria for RA. After searching the electronic medical records, we found a total of 624 patients with ICD-10 codes for RA. Among these patients, 260 were undergoing DMARD therapy at the time of first visit, 80 were diagnosed with undifferentiated arthritis and 43 were not diagnosed with inflammatory arthritis. Of the remaining 241 patients, 40 were negative for both RF and ACPA (SNRA group), and 201 were positive for either RF or ACPA (SPRA group). The flow diagram of this study is presented in Fig 1. All the patients were treated with conventional DMARDs by the rheumatologists in each hospital.

### Data collection

The data of SNRA and SPRA patients were compared as follows: 1) baseline patient characteristics such as age, sex and symptom duration; 2) disease activity measures including erythrocyte sedimentation rate (ESR), C-reactive protein (CRP), number of swollen and tender joints in the 28 joints (28 SJC and 28 TJC respectively), proportion of patients with disease activity score-28 with ESR (DAS28-ESR) remission (DAS28 < 2.6), and DAS28-ESR measured at baseline, 1 and 2 years after treatment with DMARDs. In the case of DAS28-ESR, differences from baseline to 1 and 2 years were also obtained; 3) radiographic assessment of the joints at baseline for joint space narrowing or bone erosion, and determination of patient progress radiologically by comparing baseline and follow-up radiographs in available cases; and 4) detailed drug prescription.

### Statistical analysis

Using SPSS version 20.0 (IBM Corp., Armonk, NY, USA), we used independent two-sample t-test to compare continuous variables with normal distribution. The Mann Whitney U test was used to compare continuous variables with non-normal distribution. The Chi-square test was used to assess the association between categorical variables. Disease activity measures evaluated over a 2-year period were compared by linear mixed model. In all the analyses, a *p* value of <0.05 was considered to be statistically significant.

### Ethics

This study was approved by the institutional review board (IRB) of Dongguk University Ilsan Hospital and Chung-Ang University Hospital. Informed consent was waived by the IRB due to the retrospective nature of this study.

## Results

### Baseline patient characteristic

As shown in Table 1, SPRA and SNRA patients were similar in age, sex and disease duration though SNRA patients had numerically shorter disease duration. RF and ACPA were positive in more than 95% of SPRA patients.

**Table 1:** Baseline patient characteristics.

*	SPRA (n = 201)	SNRA (n = 40)	*p* value
Age (mean±SD, years)	56.5±15.1	58.2±14.5	0.513
Female [n(%)]	149 (74.1)	27 (67.5)	0.436
Disease duration (mean±SD, months)	11.1±25.1	6.3±15.5	0.252
Follow-up duration (mean±SD, months)	31.6±21.9	27.1±24.3	0.248
RF positive [n(%)]	190 (94.5)		
ACPA positive [n(%)]	193 (96)		
Proportion of patients meeting 1987 criteria	118 (58.7)	39 (97.5)	<0.001
Proportion of patients meeting 2010 criteria	200 (99.5)	11 (27.5)	<0.001
28 TJC (mean±SD)	3.3±2.7	4.7±2.9	0.004
28 SJC (mean±SD)	2.9±2.3	4.3±3.0	0.001
ESR (mean±SD, mm/hr)	55.2±32.2	55.9±39.6	0.903
CRP (mean±SD, mm/hr)	2.5±3.9	3.6±5.2	0.135
DAS28-ESR (mean±SD)	4.7±1.0	5.1±1.0	0.043

Statistical method:

*Student’s t-test, SPRA: seropositive rheumatoid arthritis, SNRA: seronegative rheumatoid arthritis, SD: standard deviation, RF: rheumatoid factor, ACPA: anti-cyclic citrullinated peptide antibody, TJC: tender joint count, SJC: swollen joint count, ESR: erythrocyte sedimentation area, CRP: C-reactive protein, DAS28-ESR: disease activity score 28 base on ESR value

While most of the SPRA patients (200/201) fulfilled 2010 criteria for RA, most of the SNRA patients (39/40) were diagnosed according to 1987 criteria. Disease activity measures at baseline, 28 TJC (4.7±2.9 vs. 3.3±2.7, *p* = 0.004), 28 SJC (4.3±3.0 vs. 2.9±2.3, *p* = 0.001), and DAS28-ESR (5.1±1.0 vs. 4.7±1.0, *p* = 0.043) were significantly higher in SNRA patients compared with those of SPRA patients. This finding became more noticeable when these values were compared between SNRA patients fulfilling 2010 ACR/EULAR criteria and SPRA patients (Table 2.)

**Table 2:** Subgroup comparisons of baseline disease activities among SNRA patients grouped according to different diagnostic criteria (1987 ACR criteria vs. 2010 ACR/EULAR criteria) and SPRA patients.

*	**SPRA (n = 201)**	**1987 SNRA (n = 29)**	*p* value
28 TJC (mean±SD)	3.3±2.7	3.6±1.9	0.555
28 SJC (mean±SD)	2.9±2.3	3.2±1.7	0.444
ESR (mean±SD, mm/hr)	55.2±32.2	52.0±34.6	0.619
CRP (mean±SD, mm/hr)	2.5±3.9	3.2±4.7	0.387
DAS28-ESR (mean±SD)	4.7±1.0	4.9±0.8	0.588
**	**SPRA (n = 201)**	**2010 SNRA (n = 11)**	*p* value
28 TJC [median (IQR)]	3.0 (2.0~4.0)	8.0 (4.0~10.0)	<0.001
28 SJC [median (IQR)]	2.0 (1.0~4.0)	8.0 (4.0~10.0)	<0.001
ESR [median (IQR), mm/hr]	49.0 (29.5~80.0)	48.0 (31.0 ~ 120.0)	0.739
CRP [median (IQR), mg/dL]	0.9 (0.3~3.3)	2.5 (1.1~6.9)	0.047
DAS28-ESR [median (IQR)]	4.7 (4.1~5.5)	6.1 (4.8~6.4)	0.006

Statistical method

*Student’s t-test

**Mann-Whitney U test

SPRA: seropositive rheumatoid arthritis, 1987 SNRA: seronegative rheumatoid arthritis fulfilling only 1987 ACR criteria, 2010 SNRA: seronegative rheumatoid arthritis fulfilling 2010 ACR/EULAR criteria, TJC: tender joint count, SD: standard deviation, SJC: swollen joint count, ESR: erythrocyte sedimentation rate, CRP: C-reactive protein, DAS28-ESR: disease activity score 28 ESR, IQR: interquartile range

Moreover, CRP levels were significantly higher in SNRA fulfilling 2010 criteria. In contrast, there were no significant differences in baseline disease activity measures among SNRA patients fulfilling only 1987 ACR criteria and SPRA patients (Table 2). Comparison between SNRA patients fulfilling only 1987 criteria and those fulfilling 2010 criteria showed similar results demonstrating more active disease in patients fulfilling 2010 ACR/EULAR criteria (data not shown).

### Treatment outcomes

**Fig 2:**
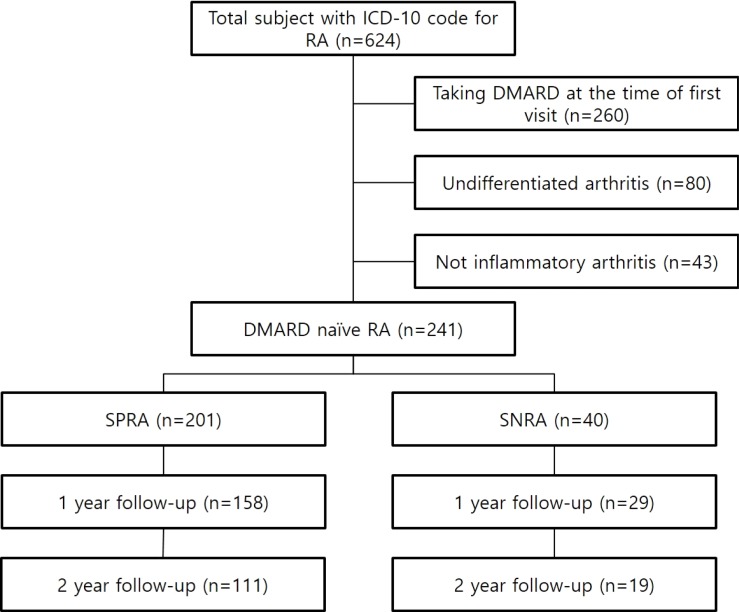
Disease activity measures (ESR, CRP, 28TJC, 28SJC and DAS28-ESR) all improved in both groups over the 2 years of treatment with DMARDs. Box plots for each disease activity measures at baseline, 1 year and 2 years after are displayed for each group (SNRA and SPRA). All the measures improved from baseline on both groups after treatment with DMARDs. DMARD: disease modifying antirheumatic drug, ESR: erythrocyte sedimentation rate, CRP: C-reactive protein, TJC: tender joint count, SJC: swollen joint count, DAS28-ESR: disease activity score 28 ESR.

Over the two years of DMARD treatment, all the disease activity measures significantly improved from baseline in the two groups (Table 3, and Fig 2).

**Table 3:** Comparisons of treatment outcome.

∮	Group	Baseline	1 year†	2 year††	p*	p**
ESR (mean±SD, mm/hr)	SPRA	55.2±32.3	22.0±18.6	21.5±17.2	<0.001	0.883
	SNRA	55.9±39.6	20.3±23.6	20.4±23.5	<0.001	
CRP (mean±SD, mg/dL)	SPRA	2.5±3.9	1.6±4.0	1.6±3.9	0.038	0.439
	SNRA	3.6±5.2	1.5±2.4	1.3±1.9	0.041	
28 TJC (mean±SD, number)	SPRA	3.3±2.7	0.5±1.0	0.5±0.8	<0.001	0.019
	SNRA	4.7±2.9	0.4±1.1	0.5±1.2	<0.001	
28 SJC (mean±SD, number)	SPRA	2.9±2.3	0.6±1.2	0.5±0.9	<0.001	0.014
	SNRA	4.3±3.0	0.4±1.0	0.4±1.2	<0.001	
DAS28-ESR(mean±SD)	SPRA	4.7±1.0	2.5±1.0	2.5±0.9	<0.001	0.895
	SNRA	5.1±1.0	2.1±1.0	2.1±1.3	<0.001	

Statistical method: ∮linear mixed model *in-group comparison **inter-group comparison † number of patients with available 1 year followup data: SPRA (n = 158) SNRA (n = 29) †† number of patients with available 2 year followup data: SPRA (n = 111), SNRA (n = 19) ESR: erythrocyte sedimentation area, SD: standard deviation, SPRA: seropositive rheumatoid arthritis, SNRA: seronegative rheumatoid arthritis, CRP: C-reactive protein, 28 TJC: tender joint count in 28 joints assessed in disease activity score 28, 28 SJC: swollen joint count in 28 joints assessed in disease activity score 28, DAS28-ESR: disease activity score 28 base on ESR value

Although baseline disease activity was higher in the SNRA group, the SNRA patients showed a better response to treatment (ΔDAS28 from baseline at 1 year in SNRA -2.9±1.2 vs. SPRA -2.2±1.8, *p* = 0.002), which yielded similar treatment outcomes in the 2 groups at the end of follow-up. In order to adjust for the different baseline disease activities in both groups, comparison of ΔDAS28 in populations grouped by baseline disease activity determined by DAS28-ESR score (high disease activity >5.1, moderate >3.2, ≤5.1, low ≥2.6, ≤3.2) was also performed. In high disease activity population [n = 90 (SPRA 72, SNRA 18)], ΔDAS28 at 1 year was significantly greater in SNRA patients compared to SPRA patients (SPRA vs. SNRA -2.84±1.32 vs. -3.70±1.29, p = 0.037) whereas in moderate disease activity population [n = 138 (SPRA 116, SNRA 22)], it showed only a trend to be greater in SNRA patients (SPRA vs. SNRA—1.97±0.95 vs. -2.40±0.85, *p* = 0.097). Comparison among low disease activity population was not possible because none of the SNRA patients was with low disease activity. Inter-group differences over 2 years were not significant in ESR, CRP and DAS28 values, while significant in 28 TJC (*p* = 0.019) and 28 SJC (*p* = 0.014). DAS28-ESR remission rate was similar between the 2 groups (1 year: SNRA 21/29 (72.4%) vs. SPRA 95/158 (60.1%), *p* = 0.298, 2 years: SNRA 14/19 (73.7%) vs. 67/113 (59.3%), *p* = 0.311). Radiologic outcomes assessed by plain radiographs of the affected joints at baseline and follow-up were also similar. No significant differences were found between the 2 groups in terms of the proportion of patients with joint space narrowing or erosion at baseline (SNRA 5/21(23.8%) vs. SPRA 48/182 (26.4%), *p* = 1.0) and in the proportion of patients with radiologic progression (SNRA 1/4 (25%) vs. SPRA 23/51 (45.1%), *p* = 0.624, follow-up interval SNRA 26.7±19.0 vs. SPRA 34.6±18.8, *p* = 0.423).

### Details of RA treatment

We investigated the details of DMARD treatment in order to determine whether these similar outcomes resulted following similar treatment (Table 4).

**Table 4:** Comparison of medication use between SPRA and SNRA.

	SPRA	SNRA	*p* value
*DMARD combination [n(%)]	119/201 (59.2)	20/40 (50)	0.298
**Initial dose of prednisolone (mean±SD, mg)	6.0±4.3	6.0±6.5	0.880
**Total number of DMARDs used until 1^st^ remission (mean±SD)	2.3±1.2	2.1±0.9	0.525
**Total number of DMARDs used (mean±SD)	2.7±1.3	2.2±1.2	0.138
**Elapsed time to 1^st^ remission (mean±SD, months)	7.4±6.5	7.1±6.2,	0.839
**MTX dose at 1^st^ remission (mean±SD, mg/week)	14.4±3.3	15.2±3.4	0.301
*Prednisolone taper-out rate at 1 year [n(%)]	32/155 (20.6)	6/29 (20.7)	1.000
*Prednisolone taper-out rate at 2 year n(%)]	41/111 (36.9)	9/18 (50)	0.308
*Rate of biological agent use at 1 year n(%)]	14/158 (8.9)	1/29 (3.4)	0.474
*Rate of biological agent use at 2 year n(%)]	15/111 (13.5)	1/19 (5.3)	0.465

Statistical method

*chi square test

**student’s t-test

SPRA: seropositive rheumatoid arthritis, SNRA: seronegative rheumatoid arthritis, DMARD: disease modifying antirheumatic drug, SD: standard deviation, MTX: methotrexate

The proportion of patients who were initiated with the combination DMARD therapy, the starting dose of prednisolone, and the total number of DMARDs used were similar in both groups. In addition, both groups achieved their first remission with similar doses and duration of methotrexate treatment. The proportion of patients using biological agents and the rate of prednisolone tapering at 1 and 2 years were not significantly different between the 2 groups.

## Discussion

This study demonstrated that SNRA patients manifested more active disease at presentation, with better response to treatment with DMARDs compared with SPRA patients. Conventional treatment with DMARDs yielded similar outcomes in the 2 groups. This study recruited patients in the setting of a rheumatology clinical practice. We did not stipulate any inclusion criteria, however, we only selected DMARD-naïve patients, who were subsequently treated in accordance with the current consensus guidelines for RA treatment. We believe that this study showcases real-world clinical presentations and treatment outcomes of both SNRA and SPRA.

In this study, SNRA patients manifested more active disease at baseline compared with SPRA patients. This could be partly explained by the fact that 99.5% of SPRA patients met the 2010 ACR/EULAR criteria while only 27.5% of SNRA patients did. The 2010 ACR/EULAR criteria give much weight to serologic markers in order to detect patients with RA early in the disease course. Therefore, seropositive patients with only one to two involved joints could be diagnosed with RA, based on the 2010 ACR/EULAR criteria even though they failed to meet the 1987 ACR criteria. This is supported by the finding in our study that the numbers of swollen and tender joints were remarkably greater in SNRA patients fulfilling 2010 criteria compared to SPRA patients. In line with our results, Nordberg, et al reported that SNRA patients showed more inflammatory activity compared with SPRA patients and these differences reflect the higher number of involved joints required for SNRA patients to fulfill the 2010 ACR/EULAR criteria [7]. Barra, et al also reported that patients with seronegative arthritis manifested greater severity of disease activity compared with seropositive patients [11]. By contrast, several previous studies, which reported similar or worse disease in SPRA patients [5, 12, 13], which may partly be attributable to the differences in characteristics of the cohort from which the patients were recruited.

The significant difference in the proportion of patients meeting each classification criteria (1987 ACR criteria and 2010 ACR/EULAR criteria) between SNRA and SPRA patients underscores the value of both criteria when diagnosing RA, especially in seronegative patients. Although the 2010 ACR/EULAR criteria enable early detection and diagnosis of RA in the disease course, they rely heavily on serology and may fail to detect some seronegative patients, as shown in this study. This finding may also prompt the primary physicians to refer seronegative patients with severe disease more often while referring seropositive patients regardless of disease severity [14].

SNRA patients, especially those with high baseline disease activity, showed better response to treatment with a greater decrease in DAS28-ESR values compared with SPRA patients, which is consistent with previous studies [5, 12, 13]. This finding could be explained by the disease duration in SNRA patients in this study, which was numerically shorter than in SPRA patients though not statistically significant. It is well known that early diagnosis and treatment is associated with good treatment outcome in RA.

The radiologic outcomes at baseline and follow-up were similar between the two groups in this study. Although follow-up radiographs were obtained in only a few patients in this study, both SPRA and SNRA patients showed similar radiological damage at baseline. This suggests that SNRA patients, if not treated properly, may have similar risks of having erosive disease as SPRA patients. In line with this finding, several recent studies reported that SNRA patients had similar or even more erosive disease at baseline compared with SPRA patients [7, 11], while several earlier studies reported the association of seropositivity with erosive disease [5, 12, 13, 15].

Although SNRA patients showed better treatment response in this study, they manifested more active disease and comparable radiological damage at baseline compared with SPRA patients. In addition, both groups received similar treatment and attained similar outcomes at 1 and 2 years irrespective of the autoantibody status. Therefore, SNRA may not be a benign subtype of RA at least in the early phase of disease and it is important not to underestimate the clinical burden of SNRA despite recent treatment guidelines justifying earlier intensive treatment in SPRA patients compared with SNRA patients [16].

Our study has several limitations. First, this study was retrospective in nature with a risk of selection bias. Second, the number of patients with SNRA was comparatively small due to the small proportion of SNRA in RA. However, the proportion of patients with SNRA among the RA patients in this study was similar to that of SNRA in general, which suggests that this study reflects the real-world scenario. Finally, the radiographs were not assessed with a validated and standard method because the radiographs were usually taken only at the affected joints. Many cases lacked radiographs needed for standard assessment tools such as Sharp van der Heijde score. Follow-up radiographs were available in only a few patients, which may increase the risk of bias and reduce generalizability.

## Conclusion

SNRA patients have more active disease and similar radiographic damage at presentation compared with SPRA patients. Although SNRA patients showed better response to treatment, physicians should be aware of the considerable clinical burden of SNRA, especially at disease onset.

## Supporting information

S1 Data Raw data. This is the raw data of this study.
(SAV)
