# Correction: Clinical management of seronegative and seropositive rheumatoid arthritis: A comparative study

**DOI:** 10.1371/journal.pone.0199468

**Published:** 2018-06-18

**Authors:** Sangtae Choi, Kwang-Hoon Lee

The first author's name is spelled incorrectly. The correct name is: Sangtae Choi. The correct citation is: Choi S, Lee K-H (2018) Clinical management of seronegative and seropositive rheumatoid arthritis: A comparative study. PLoS ONE 13(4): e0195550. https://doi.org/10.1371/journal.pone.0195550

The first author, Sangtae Choi, is incorrectly listed as the Corresponding Author. The second author, Kwang-Hoon Lee, is the Corresponding Author. The contact information appears correctly for Kwang-Hoon Lee.

The images for Figs 1 and 2 are incorrectly switched. The image that appears as Fig 1 should be Fig 2, and the image that appears as Fig 2 should be Fig 1. The figure captions appear in the correct order.

**Fig 1:**
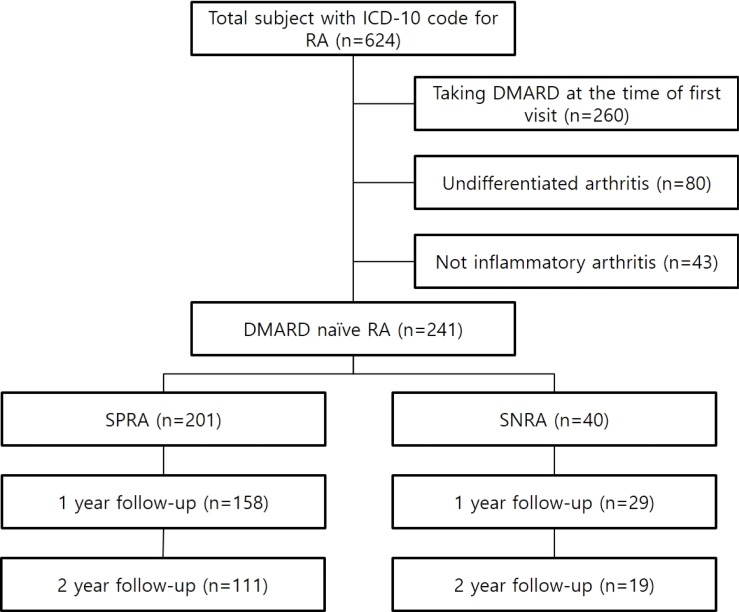
Flow diagram of the study design.

**Fig 2:**
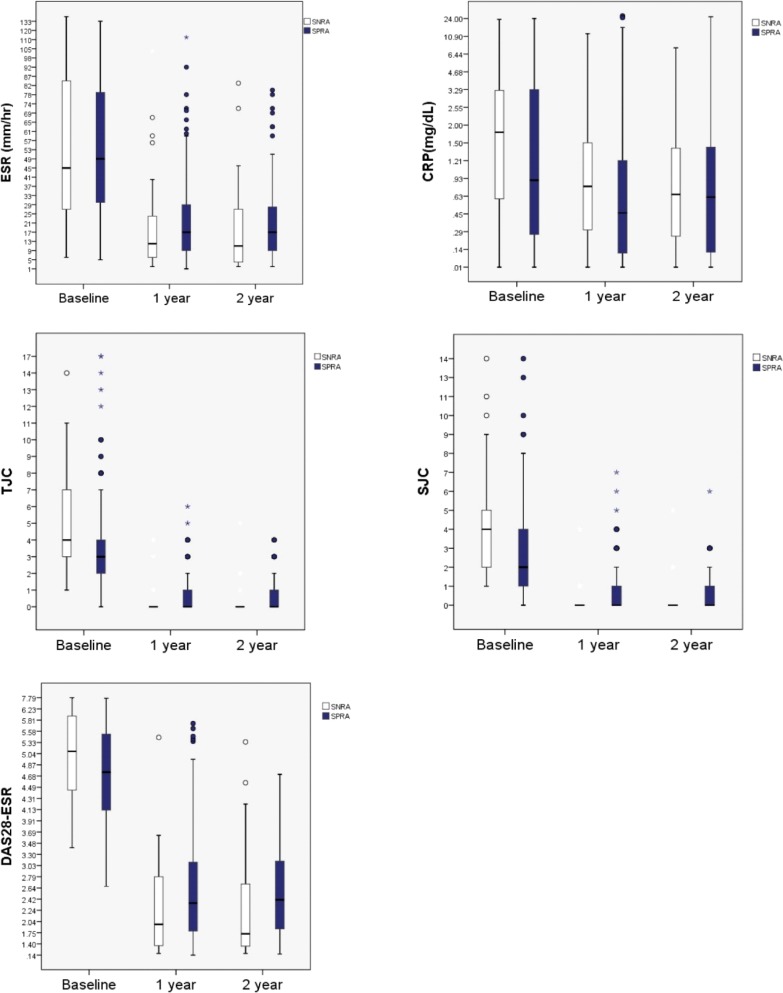
Disease activity measures (ESR, CRP, 28TJC, 28SJC and DAS28-ESR) all improved in both groups over the 2 years of treatment with DMARDs. Box plots for each disease activity measures at baseline, 1 year and 2 years after are displayed for each group (SNRA and SPRA). All the measures improved from baseline on both groups after treatment with DMARDs. DMARD: disease modifying antirheumatic drug, ESR: erythrocyte sedimentation rate, CRP: C-reactive protein, TJC: tender joint count, SJC: swollen joint count, DAS28-ESR: disease activity score 28 ESR.
